# Expression, sorting and transport studies for the orphan carrier SLC10A4 in neuronal and non-neuronal cell lines and in *Xenopus laevis* oocytes

**DOI:** 10.1186/s12868-015-0174-2

**Published:** 2015-06-19

**Authors:** Stephanie Schmidt, Marcela Moncada, Simone Burger, Joachim Geyer

**Affiliations:** Institute of Pharmacology and Toxicology, Justus Liebig University of Giessen, Schubertstr. 81, 35392 Giessen, Germany

**Keywords:** SLC10A4, Transport, NTCP, Neurotransmitter, Sorting

## Abstract

**Background:**

SLC10A4 belongs to the solute carrier family SLC10 whose founding members are the Na^+^/taurocholate co-transporting polypeptide (NTCP, *SLC10A1*) and the apical sodium-dependent bile acid transporter (ASBT, *SLC10A2*). These carriers maintain the enterohepatic circulation of bile acids between the liver and the gut. SLC10A4 was identified as a novel member of the SLC10 carrier family with the highest phylogenetic relationship to NTCP. The SLC10A4 protein was detected in synaptic vesicles of cholinergic and monoaminergic neurons of the peripheral and central nervous system, suggesting a transport function for any kind of neurotransmitter. Therefore, in the present study, we performed systematic transport screenings for SLC10A4 and also aimed to identify the vesicular sorting domain of the SLC10A4 protein.

**Results:**

We detected a vesicle-like expression pattern of the SLC10A4 protein in the neuronal cell lines SH-SY5Y and CAD. Differentiation of these cells to the neuronal phenotype altered neither *SLC10A4* gene expression nor its vesicular expression pattern. Functional transport studies with different neurotransmitters, bile acids and steroid sulfates were performed in SLC10A4-transfected HEK293 cells, SLC10A4-transfected CAD cells and in *Xenopus laevis* oocytes. For these studies, transport by the dopamine transporter DAT, the serotonin transporter SERT, the choline transporter CHT1, the vesicular monoamine transporter VMAT2, the organic cation transporter Oct1, and NTCP were used as positive control. SLC10A4 failed to show transport activity for dopamine, serotonin, norepinephrine, histamine, acetylcholine, choline, acetate, aspartate, glutamate, gamma-aminobutyric acid, pregnenolone sulfate, dehydroepiandrosterone sulfate, estrone-3-sulfate, and adenosine triphosphate, at least in the transport assays used. When the C-terminus of SLC10A4 was replaced by the homologous sequence of NTCP, the SLC10A4-NTCP chimeric protein revealed clear plasma membrane expression in CAD and HEK293 cells. But this chimera also did not show any transport activity, even when the N-terminal domain of SLC10A4 was deleted by mutagenesis.

**Conclusions:**

Although different kinds of assays were used to screen for transport function, SLC10A4 failed to show transport activity for a series of neurotransmitters and neuromodulators, indicating that SLC10A4 does not seem to represent a typical neurotransmitter transporter such as DAT, SERT, CHT1 or VMAT2.

## Background

The orphan carrier SLC10A4 belongs to the solute carrier family SLC10, whose founding members are the Na^+^/taurocholate co-transporting polypeptide NTCP (*SLC10A1*) and the apical sodium-dependent bile acid transporter ASBT (*SLC10A2*) [1]. These carriers maintain the enterohepatic circulation of bile acids by sodium-dependent bile acid reabsorption in the gut via ASBT and sodium-dependent bile acid transport into hepatocytes via NTCP [2]. A further member of this carrier family, the sodium-dependent organic anion transporter SOAT (*SLC10A6*), is expressed in spermatocytes in man and mouse and has transport activity for sulfo-conjugated steroid hormones [3–5]. The other members of this carrier family, SLC10A3, SLC10A4, SLC10A5, and SLC10A7, have been characterized at the molecular and expression level, but still remain orphan carriers [1, 6–12].

The SLC10A4 transcript was first cloned from rat in our group in 2004 and showed predominant expression in the central nervous system in man, rat, and mouse [13]. Further expression analyses on the protein level localized SLC10A4 to cholinergic and monoaminergic neurons of the central and peripheral nervous system as well as to mast cells [13–15]. In contrast to NTCP, ASBT, and SOAT, the SLC10A4 protein is not directed to the plasma membrane, but typically showed a vesicular expression pattern in neuronal cells, mast cells, and even in transiently or stably transfected cells lines [6, 13–15]. Based on this expression pattern, it was assumed that SLC10A4 may represent a novel vesicular carrier for any kind of neurotransmitter or neuromodulator [1, 6, 13–15]. More recently, in a series of very elegant experiments on *Slc10a4* knockout mice it was shown by the Kullander group that these mice are hypersensitive to the psychostimulants amphetamine and tranylcypromine, and have an altered response to cholinergic stimuli at the neuromuscular junction and in the central cholinergic system, suggesting that SLC10A4 may contribute to the vesicular storage or release of neurotransmitters [15–17]. Therefore, in the present study, we performed systematic transport screenings for SLC10A4 in transfected neuronal and HEK293 cell lines as well as in *Xenopus laevis* oocytes and also aimed to identify the vesicular sorting domain of the SLC10A4 protein. Although we have not identified a transported substrate for SLC10A4 to date, recent descriptions of taurocholic acid and lithocholic acid transport by a thrombin-modified variant of SLC10A4 [18] encouraged us to present our data to provide a broader basis for further SLC10A4 transport studies.

## Results

### Endogenous expression of SLC10A4 in neuronal cell lines

The primary goal of the present study was to identify a transported substrate for the orphan carrier SLC10A4 with an in vitro approach. As the endogenous expression of SLC10A4 is exclusively directed to neuronal cells and mast cells [13, 14], neuronal cell cultures were thought to be the most appropriate for this purpose. Therefore, we analyzed SLC10A4 expression in the human neuroblastoma cell line SH-SY5Y as well as in the mouse cell line CAD (Cath.a-differentiated neuronal cells, originating from the locus coeruleus in the brainstem) with different SLC10A4-directed antibodies.

**Figure 1:**
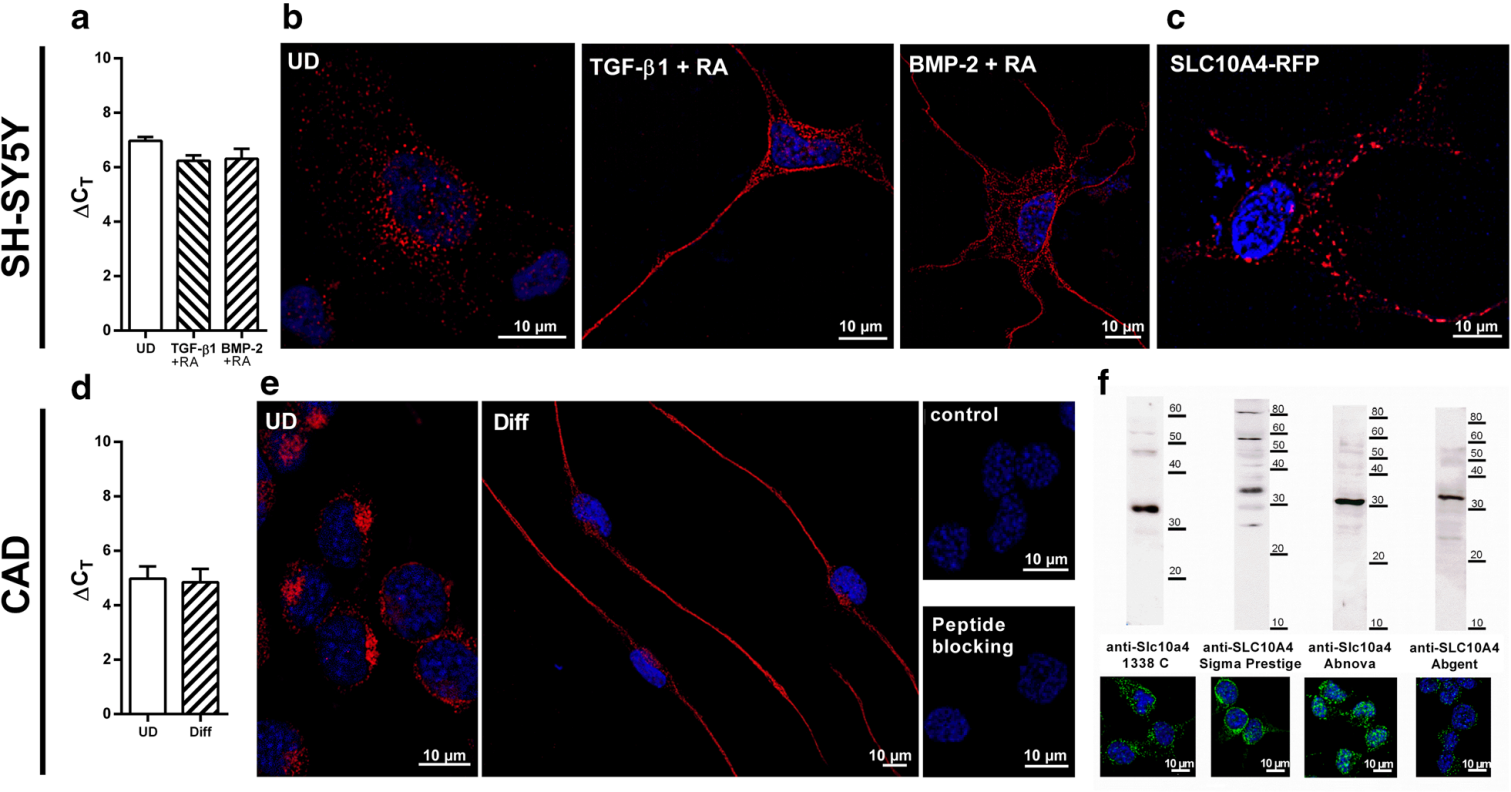
Expression and subcellular localization of SLC10A4 in SH-SY5Y and CAD cells. **a** Relative *SLC10A4* gene expression in SH-SY5Y cells after differentiation with TGF-ß1 + RA or BMP-2 + RA. Values represent mean ± SD of triplicate measurements. **b** Immunofluorescence analysis of the subcellular expression of the SLC10A4 protein in SH-SY5Y cells. Cells were either untreated (UD), or were differentiated with TGF-ß1 + RA or BMP-2 + RA over 4 days prior to immunolabeling. The SLC10A4 protein was detected with the anti-Slc10a4 1338 C antibody (1:1,000) and the Cy3-labelled anti-rabbit secondary antibody (1:800, red fluorescence) and nuclei were stained with DAPI (blue fluorescence). In all cases, the SLC10A4 protein showed a vesicle-like expression pattern within the perikarya and along the neurite-like cellular protrusions. **c** Even when a fluorescence-tagged SLC10A4-RFP construct was transiently transfected into SH-SY5Y cells, the SLC10A4-RFP protein showed a clear vesicular sorting pattern. **d** Relative *Slc10a4* gene expression analysis in differentiated (Diff) and undifferentiated (UD) CAD cells. The values represent mean ± SD of triplicate measurements. **e** Endogenous expression of the SLC10A4 protein in CAD cells, cultivated in FCS containing medium (UD) or FCS-free medium (Diff). The SLC10A4 protein was detected with the anti-Slc10a4 1338 C antibody (1:500) and the Cy3-labelled secondary antibody (1:800, red fluorescence). For control, the primary anti-Slc10a4 antibody was omitted (control) or the antibody was pre-incubated with the immunizing peptide (peptide blocking). **f** Immunofluorescence detection of the SLC10A4 protein was performed with different SLC10A4-directed antibodies (green fluorescence): self-generated polyclonal rabbit anti-Slc10a4 1338 C antibody, rabbit anti-SLC10A4 Sigma Prestige antibody, rabbit anti-Slc10a4 Abnova antibody, and rabbit anti-SLC10A4 Abgent antibody. Membrane protein enriched fractions of the CAD cells were also subjected to Western Blot analysis with the same antibodies and revealed specific bands for the SLC10A4 protein at an apparent molecular weight of 30–32 kDa.

SH-SY5Y cells showed a typical neuroblast-like appearance with small, round cell bodies and occasional short extensions. Under incubation with retinoic acid (RA) and the neurotropic factors tumor growth factor beta (TGF-β1) and bone morphogenetic protein 2 (BMP-2), the cells stopped proliferation and developed neurite-like long extensions, as described previously [19, 20]. Under both conditions, SLC10A4 showed a clear vesicle-like expression pattern in the SH-SY5Y cells and was detectable even along the long neurite-like outgrowths, indicating sorting of the SLC10A4 protein to the synaptic direction of the differentiated SH-SY5Y cells (Figure 1b). At the RNA level, SLC10A4 showed an overall higher expression in the SH-SY5Y cells compared with vesicular acetylcholine transporter (VAChT) and vesicular monoamine transporter 2 (VMAT2) (data not shown), but incubation with TGF-β1 + RA or BMP-2 + RA did not significantly affect the SLC10A4 mRNA expression levels, indicating that *SLC10A4* expression is not regulated by the RA, BMP-2, or TGF-β1 triggered signaling cascades (Figure 1a). Although transient transfection of SLC10A4 into SH-SY5Y revealed an identical expression pattern compared with the endogenous expression, as shown for an SLC10A4-RFP construct in Figure 1c, the transfection rate of these cells could not be enhanced above 20% by different transfection methods (lipofection, non-liposomal transfection, electroporation), meaning that SH-SY5Y cells overexpressing SLC10A4 vs. non-transfected SH-SY5Y cells could not be used for transport studies. For the same reason, down-regulation of SLC10A4 expression by transfection of SLC10A4 siRNA prior to transport experiments was also not considered.

Similar to the SH-SY5Y cells, the SLC10A4 protein was detected in vesicular structures in mouse CAD cells. These cells were differentiated to a neuronal phenotype by serum depletion, as previously reported [21]. As shown in Figure 1e, under these conditions, long neurite-like extensions were formed which were highly immunoreactive for the anti-Slc10a4 antibody. However, even in these cells, RNA expression of SLC10A4 was not significantly affected by neuronal differentiation (Figure 1d). Although CAD cells could easily be transfected with different SLC10A4 constructs with transfection rates above 80%, these cells significantly detached after the transfection procedure and then could not be further subjected to standard transport assays. Therefore, overexpression of SLC10A4 or down-expression by RNAi transfection of these cells was not practicable for transport screening assays.

Localization of the SLC10A4 protein in the central and peripheral nervous system of the rat, as well as in rat PC12 cells [13, 14], human SH-SY5Y cells, and mouse CAD cells (present study), was performed by us with a self-generated polyclonal rabbit antibody—1338 C—directed against the amino acid residues 422–437 (VGTDDLVLMETTQTSL) of the deduced rat Slc10a4 protein sequence [GenBank:AAV80706]. In order to further verify SLC10A4 expression in neuronal cell cultures with different antibodies, we screened mouse CAD cells by immunofluorescence and Western blot analysis, and found by all of the used antibodies—directed against the 324–437 amino acids of the C-terminus of human SLC10A4 protein (Sigma Prestige rabbit anti-SLC10A4), the 1–27 amino acids of the N-terminus of mouse and rat SLC10A4 protein (Abnova rabbit anti-Slc10a4), and the 377–406 amino acids of the C-terminus of the human SLC10A4 protein (Abgent rabbit anti-SLC10A4)—a clear vesicle-like expression pattern. The mouse SLC10A4 protein consistently showed an apparent molecular weight of 30–32 kDa in the Western blot (Figure 1f). This is below the calculated molecular weight of 47 kDa, which has been reported for other members of the SLC10 carrier family as well [8].

### In vitro transport studies on SLC10A4 with candidate substrates

**Figure 2:**
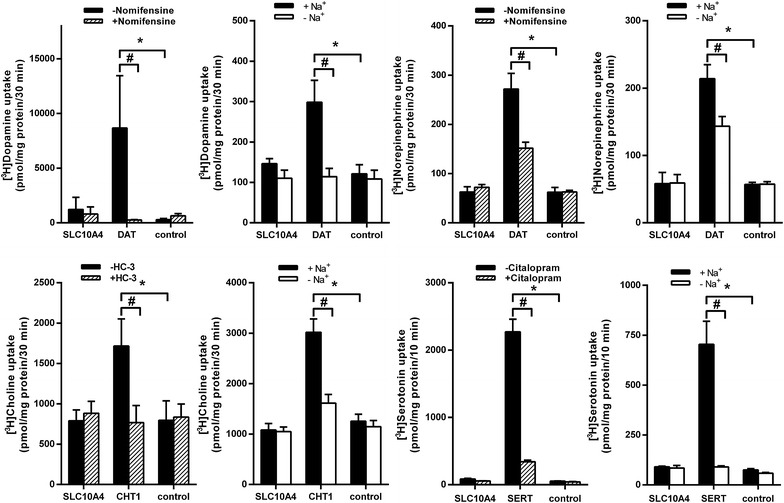
Transport measurements in transiently transfected HEK293 cells. HEK293 cells were transiently transfected with the indicated carriers SLC10A4, DAT, CHT1, or SERT, respectively. The uptake of 5 µM [^3^H]dopamine, 5 µM [^3^H]norepinephrine, 5 µM [^3^H]choline, or 5 µM [^3^H]serotonin was measured over the given time periods in the presence and absence of Na^+^ (for CHT1 Na^+^ was replaced by Li^+^). Transport via DAT, CHT1, and SERT was blocked by the specific inhibitors nomifensine (10 µM), hemicholinium-3 (HC-3, 1 µM), and citalopram (2 µM), respectively. Values represent mean ± SD of representative experiments, each with quadruplicate determinations (n = 4). *Significantly different from control with *p* < 0.001. ^#^Significantly different from positive uptake, *p* < 0.001.

As these neuronal cell lines were difficult to handle for standard transport experiments, we decided to use transfected human embryonic kidney (HEK) 293 cells as well as SLC10A4 cRNA injected *X. laevis* oocytes, which are both well-established in vitro cell models for transport measurements [3, 22]. We previously reported that, apart from its predominant intracellular expression, the SLC10A4 protein can to a lower extent also be detected in the plasma membrane of transfected HEK293 cells [13]. Therefore, we first used HEK293 cells transiently transfected with the human SLC10A4 construct for classical uptake measurement in intact cells. As positive controls, the plasma membrane neurotransmitter transporters dopamine transporter (DAT), choline transporter (CHT1), and serotonin transporter (SERT) were also transfected, and empty-vector transfected cells served as negative controls. Whereas DAT showed a significant nomifensine-sensitive and sodium-dependent uptake of [^3^H]dopamine, SLC10A4-transfected HEK293 cells tended to accumulate more dopamine compared with the control, but without reaching a level of significance (Figure 2). SLC10A4 showed no transport activity for [^3^H]norepinephrine, [^3^H]serotonin and [^3^H]choline at all, whereas DAT, SERT and CHT1 significantly transported their substrates in a sodium-dependent manner.

**Figure 3:**
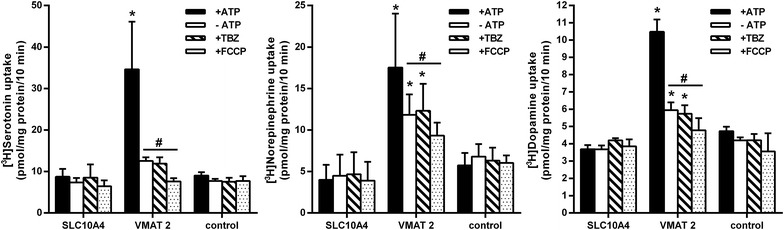
Transport measurements in digitonin permeabilized SLC10A4-HEK293 and VMAT2-HEK293 cells. Prior to transport measurements, stably transfected human SLC10A4-HEK293 cells and human VMAT2-HEK293 cells were pre-incubated with 15 µM digitonin for 10 min for permeabilization. Then the uptake of 400 nM [^3^H]serotonin, [^3^H]norepinephrine, or [^3^H]dopamine was measured over 10 min in the presence and absence of 5 mM ATP in the transport buffer. In addition to ATP, 2 µM of the potent VMAT2 inhibitor tetrabenazine (TBZ) or 5 µM of the proton ionophore carbonylcyanide-p-trifluoromethoxyphenylhydrazon (FCCP) were added to the transport buffer as indicated. After 10 min, the cells were washed with ice-cold PBS, lysed, and subjected to scintillation counting. The values represent mean ± SD of one representative experiment (for dopamine, n = 4) or two independent experiments (for serotonin and norepinephrine, n = 8). *Significantly different from control with *p* < 0.05. ^#^Significantly different from positive uptake, *p* < 0.05.

In our previous study, we reported co-expression of the SLC10A4 protein with VMAT2 in synaptic vesicles of rat brain preparations [14], so we next intended to analyze the SLC10A4 transport in direct comparison with VMAT2. In this approach, we used HEK293 cells stably transfected with human SLC10A4 or human VMAT2, which were permeabilized with digitonin in order to facilitate access of the transport substrate to the site of carrier expression in intracellular vesicular structures, as described before [23]. As shown in Figure 3, VMAT2 showed significant transport activity for [^3^H]serotonin, [^3^H]norepinephrine, and [^3^H]dopamine which was dependent on the presence of adenosine triphosphate (ATP) in the transport buffer as well as sensitive to tetrabenazin (TBZ) and carbonylcyanide-p-trifluoromethoxyphenylhydrazon (FCCP). However, in contrast, SLC10A4 showed no transport activity for serotonin, norepinephrine, and dopamine at all in this vesicular transport assay. We also could not demonstrate a transport of acetylcholine, neither for SLC10A4 nor for VAChT in permeabilized HEK293 cell lines transfected with the respective construct (data not shown).

**Figure 4:**
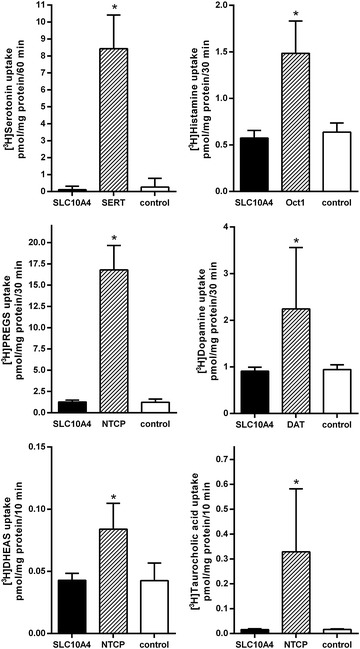
Transport measurements in *Xenopus laevis* oocytes. *Xenopus laevis* oocytes were injected with cRNA coding for human SLC10A4, SERT, DAT, or NTCP as well as mouse Oct1. Uptake of [^3^H]serotonin, [^3^H]histamine, [^3^H]PREGS, [^3^H]dopamine, [^3^H]DHEAS, or [^3^H]taurocholic acid, each at 1 µM, was measured over a time period of 10–60 min as indicated in the presence of sodium chloride in the transport buffer. SERT, Oct1, and DAT served as controls for the transport of [^3^H]serotonin, [^3^H]histamine, and [^3^H]dopamine, respectively. NTCP was the reference carrier for PREGS, DHEAS and taurocholic acid. Afterwards, the oocytes were washed with ice-cold transport buffer, lysed and subjected to scintillation counting. The values represent mean ± SD of one representative experiment with n = 10 oocytes each. *Significantly different from control with *p* < 0.001.

In previous studies at our institute we learned that the transport rates for a particular carrier may be higher in the *X. laevis* oocytes expression system compared with transfected cell cultures. Furthermore, we demonstrated in a previous study that at least the rat SLC10A4 protein is expressed at the plasma membrane of *X. laevis* oocytes [13]. Therefore, we injected SLC10A4 cRNA in *X. laevis* oocytes and screened for transport activity with a series of neurotransmitters and further SLC10A4 candidate substrates. For these experiments, water-injected oocytes served as negative control and, if appropriate, oocytes injected with cRNA coding for SERT, DAT, NTCP, or mouse organic cation transporter 1 (Oct1) were used as positive controls. As shown in Figure 4, SERT, DAT, and Oct1 showed significant transport activity for serotonin, dopamine, and histamine, respectively, but SLC10A4 cRNA injected oocytes were not different from the control. Furthermore, the neurosteroids pregnenolone sulfate (PREGS) and dehydroepiandrosterone sulfate (DHEAS) as well as the bile acid taurocholic acid, were not transported by SLC10A4 in this transport assay, although NTCP showed significant transport activity for all of them. Apart from these candidate substrates, SLC10A4 also showed no transport activity for aspartate, acetate, choline, gamma-aminobutyric acid (GABA), norepinephrine, glutamate, acetylcholine, estrone-3-sulfate (E-3-S), lithocholic acid, and ATP in the *X. laevis* oocytes model (Table 1).

**Table 1:** Transport studies in *Xenopus laevis* oocytes

	SLC10A4	Control	Ratio
*Uptake (pmol/oocyte/60 min)*			
[^3^H]Histamine (10 µM)	0.29 ± 0.02	0.27 ± 0.01	1.0
[^3^H]Aspartate (1 µM)	0.22 ± 0.01	0.28 ± 0.01	0.8
[^3^H]Acetate (25 µM)	8.79 ± 0.51	8.36 ± 0.72	1.0
[^3^H]Choline (1 µM)	2.68 ± 0.52	2.20 ± 0.19	1.2
[^3^H]GABA (1 µM)	0.52 ± 0.05	0.47 ± 0.04	1.1
[^3^H]Norepinephrine (1 µM)	0.16 ± 0.01	0.21 ± 0.02	0.8
[^3^H]Glutamate (1 µM)	0.014 ± 0.001	0.014 ± 0.002	1.0
*Uptake (pmol/oocyte/30 min)*			
[^3^H]Acetylcholine (10 µM)	43.51 ± 3.62	37.51 ± 3.67	1.2
[^3^H]Dopamine (10 µM)	1.36 ± 0.06	1.43 ± 0.05	1.0
[^3^H]PREGS (1 µM)	1.19 ± 0.09	1.06 ± 0.09	1.1
[^3^H]DHEAS (1 µM)	0.043 ± 0.002	0.043 ± 0.004	1.0
[^3^H]E-3-S (1 µM)	0.050 ± 0.001	0.060 ± 0.005	0.8
*Uptake (pmol/oocyte/10 min)*			
[^3^H]Lithocholic acid (0.3 µM)	3.68 ± 0.23	4.13 ± 0.07	0.9
[^3^H]Taurocholic acid (0.3 µM)	0.0158 ± 0.0009	0.0159 ± 0.0007	1.0
[^3^H]ATP (5 mM)	70.66 ± 9.06	68.84 ± 8.26	1.0
[^35^S]Adenosine 5`-(γ-thio) triphosphate (5 mM)	69.76 ± 4.36	67.73 ± 2.53	1.0

*Xenopus laevis* oocytes were injected with human SLC10A4 cRNA or with water (control). Values represent uptake of the indicated compound as mean ± SD of at least 10 oocytes per group. Ratio represents uptake into SLC10A4 cRNA-injected oocytes divided by uptake into water-injected oocytes.

**Figure 5:**
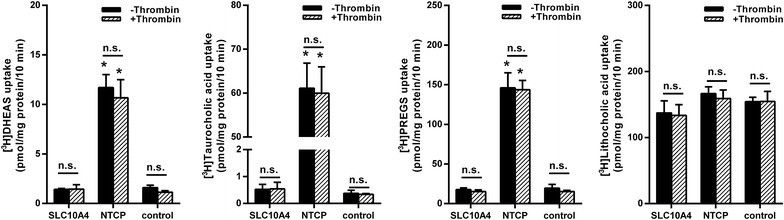
Transport measurements in stably transfected SLC10A4-HEK293 and NTCP-HEK293 cells after thrombin treatment. For the transport measurements, one part of the cells was pre-incubated with 1 U/200 µl thrombin over 3 h (+Thrombin), before the uptake of [^3^H]DHEAS, [^3^H]taurocholic acid, [^3^H]PREGS, or [^3^H]lithocholic acid (each at 300 nM) was measured over a time period of 10 min at 37°C. The cells were washed with ice-cold PBS, lysed, and subjected to scintillation counting. The values represent mean ± SD of two independent experiments each with triplicate determinations. *Significantly different from control with *p* < 0.001;* n.s.* not significantly different.

Very recently, it was reported that the N-terminal domain of the SLC10A4 protein could be cleaved by thrombin treatment and thereby transport activity for taurocholic acid and lithocholic acid may be activated [18]. Although these data were obtained in a neuronal cell culture of TE671 cells, in the present study, we intended to reproduce these data on the isolated over-expressed SLC10A4 protein and used HEK293 cells stably transfected with SLC10A4 as well as with NTCP for the control. Both, SLC10A4-HEK293 and NTCP-HEK293 cell lines were treated with 1 U/200 µl of thrombin for 3 h prior to transport measurements with [^3^H]DHEAS, [^3^H]taurocholic acid, [^3^H]PREGS, and [^3^H]lithocholic acid. As expected, NTCP showed significant transport activity for DHEAS, taurocholic acid and PREGS, and was not affected by thrombin treatment. In contrast, SLC10A4 did not transport any of these compounds, even after pre-treatment with thrombin (Figure 5). No transport activity could be observed for lithocholic acid at all, neither in NTCP nor in the SLC10A4-expressing HEK293 cells. Therefore, transport activity of SLC10A4 could not be activated by thrombin treatment in the cell model used.

### Localization of the vesicular sorting domain in the C-terminus of SLC10A4

**Figure 6:**
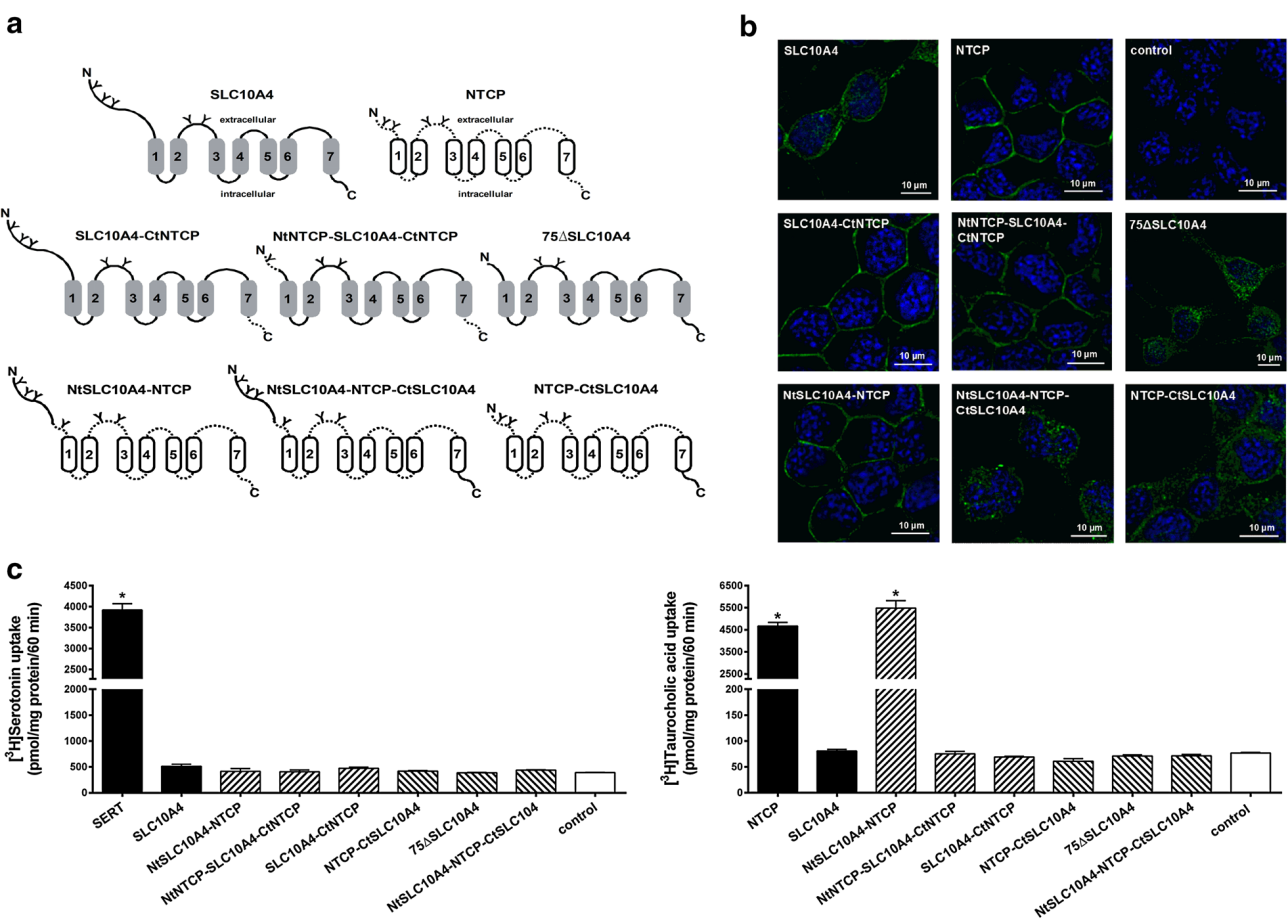
Localization and transport function of SLC10A4/NTCP chimeras in CAD cells. **a** The shown SLC10A4/NTCP chimeric constructs were used. All chimeras were generated based on the full length sequences of SLC10A4 (grey marked transmembrane domains and loops with continuous lines) and NTCP (white transmembrane domains and loops as dotted lines), both with c-terminal V5-tag. Potential glycosylation sites were marked by “Y”. **b** All constructs were transiently transfected in CAD cells and cellular localization was analyzed by immunofluorescence microscopy using rabbit anti-V5 antibody and donkey Cy3-labelled anti-rabbit secondary antibody. Nuclei were stained with DAPI. Whereas SLC10A4 showed a clear vesicle-like expression pattern, the immunofluorescence signals for NTCP, NtSLC10A4-NTCP, SLC10A4-CtNTCP, and NtNTCP-SLC10A4-CtNTCP were clearly directed to the plasma membrane. When the 75 N-terminal amino acids were deleted in SLC10A4, the 75ΔSLC10A4 protein retained its vesicle-like intracellular expression comparable with full-length SLC10A4. **c** The SLC10A4/NTCP chimeras were also used for transport studies after transient transfection into CAD cells with [^3^H]taurocholic acid and [^3^H]serotonin, each at 5 µM. These measurements were performed by incubating the cells for 60 min at 37°C in 250 µl cell medium with 50 µl sodium transport buffer containing the radiolabeled and non-radiolabeled compounds. NTCP and SERT were used as a positive control, and empty-vector transfected cells served as the negative control. After the uptake phase, cells were washed with ice-cold PBS, lysed, and subjected to scintillation counting. Data represent mean ± SD of representative experiments each with quadruplicate determinations. *Significantly different from control with *p* < 0.01.

A further aim of the present study was to localize the particular domain of SLC10A4 that is responsible for the vesicular sorting of this protein. This question was of particular interest, as mutation of this domain might redirect the SLC10A4 protein to the plasma membrane, which would facilitate further substrate screening. As within the SLC10 carrier family, NTCP, which is expressed at the plasma membrane, is the most related carrier to SLC10A4, we generated several SLC10A4/NTCP chimeric constructs in which the C-terminal and N-terminal domains were interchanged between both carriers (Figure 6a). Furthermore, we generated a 75ΔSLC10A4 mutant which was truncated by the first 75 N-terminal amino acids. All constructs were transfected in neuronal CAD cells as well as in HEK293 cells and used for immunofluorescence analysis of protein localization (Figure 6b) as well as for transport experiments with SLC10A4 candidate substrates (Figure 6c; Table 2). As shown in Figure 6b, NTCP showed clear plasma membrane localization in CAD cells, whereas SLC10A4 showed expression in intracellular vesicles. Interestingly, both chimeras bearing the C-terminus of NTCP (i.e. SLC10A4-CtNTCP and NtNTCP-SLC10A4-CtNCTP) were detected at the plasma membrane, whereas all chimeric proteins with the C-terminus of SLC10A4 (i.e. NTCP-CtSLC10A4, NtSLC10A4-NTCP-CtSLC10A4) were detected in intracellular vesicles. This sorting pattern was basically the same when these constructs were transfected and analyzed in HEK293 cells (data not shown). This indicates that the C-termini of NTCP and SLC10A4 are dominant for the sorting to the plasma membrane and intracellular vesicles, respectively. In contrast, truncation of the SLC10A4 N-terminus (75ΔSLC10A4 mutant) as well as transfer of the SLC10A4 N-terminus into the NTCP (NtSLC10A4-NTCP chimera) did not affect the sorting of SLC10A4 and NTCP, respectively, indicating that the C-terminus, but not the N-terminus of the SLC10A4 protein is relevant for the protein sorting. Apart from localizing the sorting domains in the SLC10A4 protein, several of the constructs were of particular interest for transport experiments. (I) When the N-terminal domain of SLC10A4, which was previously supposed to hinder substrate binding to the SLC10A4 carrier protein [18], were transferred to the NTCP in order to produce a NtSLC10A4-NTCP elongated chimera, the transport function for taurocholic acid remained completely intact compared with wild-type NTCP (Figure 6c). (II) Furthermore, activation of the SLC10A4-mediated taurocholic acid transport by thrombin cleavage of the N-terminus was supposed [18]. However, for the N-terminally truncated 75ΔSLC10A4 protein, as well as for the NtNTCP-SLC10A4-CtNTCP chimera, still no transport activity for taurocholic acid could be observed. (III) Just by replacing the SLC10A4 C-terminal 63 amino acids by the corresponding C-terminus of NTCP in the SLC10A4-CtNTCP and NtNTCP-SLC10A4-CtNTCP chimera, the SLC10A4 protein was directed to the plasma membrane. Therefore, these chimeras represent an interesting tool for SLC10A4 substrate screening. As the CAD cells detached after transfection of the respective constructs when they were further processed for the routine transport assay, the radiolabelled candidate substrates were added directly into the cell culture medium and cell associated radioactivity was analyzed. However, neither SLC10A4-CtNTCP nor NtNTCP-SLC10A4-CtNTCP showed transport activity so far for taurocholic acid and serotonin (Figure 6c) as well as for DHEAS, PREGS, E-3-S, dopamine, glutamate, histamine, acetylcholine, and choline (Table 2).

**Table 2:** Transport studies in HEK293 cells

Compound (5 µM)	NTCP		SLC10A4		NtNTCP-SLC10A4-CtNTCP	
	Uptake	Ratio	Uptake	Ratio	Uptake	Ratio
[^3^H]E-3-S	276.93 ± 29.12	10.1*	35.23 ± 2.01	1.3	27.91 ± 1.38	1.0
[^3^H]DHEAS	225.12 ± 16.51	6.9*	38.22 ± 0.90	1.2	31.84 ± 1.38	1.0
[^3^H]PREGS	1,656.09 ± 201.01	4.7*	366.06 ± 22.23	1.0	383.62 ± 17.38	1.0

	DAT		SLC10A4		NtNTCP-SLC10A4-CtNTCP	
[^3^H]Dopamine	1,578.17 ± 107.47	13.2*	128.61 ± 16.60	1.1	157.92 ± 19.11	1.3
[^3^H]Glutamate			22.60 ± 2.95	1.4	13.57 ± 1.62	0.8
[^3^H]Histamine			26.24 ± 1.40	1.3	25.68 ± 0.63	1.3
[^3^H]Acetyl-choline			352.00 ± 38.76	1.0	307.64 ± 31.41	0.9
[^3^H]Choline			929.53 ± 30.50	1.1	866.83 ± 23.63	1.0

Values represent uptake in pmol/mg protein/30 min of the indicated compound as mean ± SD of quadruplicate determinations. Ratio represents uptake into carrier-expressing HEK293 cells divided by uptake into mock-transfected HEK293 cells. * Significantly different from control with *p* < 0.01.

## Discussion

SLC10A4 is a member of the SLC10 carrier family commonly referred to as the “family of sodium-dependent bile acid transporters”. The rat Slc10a4 transcript was first cloned in our group in 2004 [GenBank:AY825923] [13] and the human SLC10A4 sequence was published in 2006 [6]. The SLC10A4 protein consists of 437 amino acids in man, rat, and mouse, and shows a close phylogenetic relationship to the sodium-dependent bile acid transporter NTCP. However, whereas NTCP is specifically expressed at the sinusoidal plasma membrane of hepatocytes [2], the SLC10A4 protein was localized in cholinergic and monoaminergic neurons of the central and peripheral nervous system as well as in pheochromocytoma PC12 cells and mast cells [13–15]. Based on its genetic classification, SLC10A4 first was suggested to be a novel carrier for already established substrates of the SLC10 carrier family, including bile acids and sulfo-conjugated steroid hormones [1, 6, 13]. However, based on its expression pattern, it seems more likely that SLC10A4 plays a role for the vesicular storage or exocytosis of any kind of neurotransmitter or mediator in neurons and mast cells [14]. In previous experiments, the neurosteroids PREGS and DHEAS were already considered to be particularly promising candidate substrates. These neurosteroids can modulate several postsynaptic receptor systems and even interfere with the release of multiple neurotransmitters including acetylcholine, norepinephrine, dopamine and serotonin [24–26]. However, until now, all transport measurements performed with E-3-S, DHEAS, and PREGS have so far failed to show transport function for SLC10A4 [6, 13] (present study). However, a more systematic transport screening also including classical neurotransmitters as candidate substrates has not yet been performed. Therefore, in the present study, we aimed to screen for SLC10A4 transport function with different transport assays, including transport in intact HEK293 cells and neuronal CAD cells, permeabilized HEK293 cells and *X. laevis* oocytes. Overall, no transport activity for SLC10A4 was found for taurocholic acid, lithocholic acid, DHEAS, PREGS, E-3-S, acetylcholine, choline, acetate, glutamate, aspartate, GABA, ATP, serotonin, histamine, dopamine, and norepinephrine in the respective transport assay. Although we used different kinds of transport assays and cell models, it might be the case that SLC10A4 has transport function for one of the mentioned compounds anyway. In HEK293 cells and *X. laevis* oocytes, an additional factor might have been missed that is only present in neuronal cells. However, overexpression of the SLC10A4 protein or permeabilization of the cells prior to transport experiments was not practicable in neuronal cell lines as the cells either showed low transfection rates (as the SH-SY5Y cells) or promptly detached when the incubation medium was repeatedly changed (CAD cells). Furthermore, all transport assays performed in SLC10A4-transfected CAD cells also failed to show transport activity for SLC10A4. Nevertheless, further investigations on the SLC10A4 transport function should focus on vesicle preparations from SLC10A4-deleted neuronal cell cultures or may use liposomal reconstitution of the SLC10A4 protein.

Very recently, it was suggested by Abe et al. [18] that SLC10A4 may represent a protease-activated transporter for the bile acids taurocholic acid and lithocholic acid. They used the human medulloblastoma cell line TE671 in which they localized the SLC10A4 protein by immunofluorescence and Western Blot analysis with a commercial anti-SLC10A4 antibody and showed that pre-incubation of the cells with 1U/200 µl of thrombin for 3 h increased the cellular uptake of taurocholic acid and lithocholic acid [18]. However, appropriate controls demonstrating specificity of the antibody were not provided and the authors failed to seriously show saturable transport kinetics for this uptake. Furthermore, bioinformatics analysis (Expasy Peptide Cutter, http://www.web.expasy.org/peptide_cutter/) indicates that the mouse and rat SLC10A4 proteins can be cleaved by thrombin at amino acid position 87, but not the human SLC10A4 protein. Nevertheless, in the present study, these experiments were repeated by using HEK293 cells overexpressing SLC10A4, providing a better controllable system, but did not show any transport activity of SLC10A4 for taurocholic acid after treatment with thrombin. Due to these negative data and based on the bioinformatics prediction we did not further analyze whether the human SLC10A4 protein can really be cleaved by thrombin in our experimental setup. Furthermore, when the large N-terminus of SLC10A4 was deleted by mutagenesis in the NtNTCP-SLC10A4-CtNTCP and 75ΔSLC10A4 constructs, no transport activity for taurocholic acid was detected. This is of particular interest, because it was suggested by Abe et al. [18] that the N-terminal domain of SLC10A4 might hinder substrate binding to the protein. This suggestion can not be supported by data from the present study. It is already known that thrombin starting from 1 U/200 µl has a dose-dependent effect on cell viability of neuronal cell cultures [27]. Furthermore, it was reported that lithocholic acid selectively kills neuroblastoma cells by triggering the extrinsic and intrinsic apoptotic death pathways via binding to the surface [28]. Therefore, it cannot be excluded that the suggested uptake of taurocholic acid and lithocholic acid may just have resulted from the combined surface effects of the bile acids and thrombin, but not from carrier-mediated uptake into the cells via a thrombin-modified SLC10A4 variant.

During the completion of this study, the Kullander group published a series of very elegant experiments on *Slc10a4* knockout mice, which provide further intriguing indications of the molecular transport function of SLC10A4. In the first study by Zelano et al. [16], they analyzed whether the absence of SLC10A4 may have an impact on the function of the central cholinergic system. Injection of the cholinergic agonist pilocarpin induced status epilepticus earlier and more often in the *Slc10a4* knockout mice compared with the wild type mice, suggesting that SLC10A4 may suppress epileptiform activity. They concluded that absence of the SLC10A4 protein results in cholinergic hypersensitivity of the knockout mice [16]. In a second study by Patra et al. [17], the role of SLC10A4 on the structure and function of the neuromuscular junction was analyzed. Although there were no abnormalities detectable at the macrostructure level, the *Slc10a4* knockout mice showed misshapen neuro-muscular junctions with an increased number of isolated acetylcholine receptor clusters and a decreased number of endplate branches. Nevertheless, the knockout mice had normal motor behavior. Electrophysiological measurements on nerve-muscle preparations then showed that the *Slc10a4* knockout mice had decreased spontaneous endplate potential amplitudes, which could be explained by lower acetylcholine release at the endplates without nerve stimulation. On the other hand, after repeated stimulation, the *Slc10a4* knockout mice revealed an enlarged pool of readily releasable vesicles at the neuro-muscular junction. However, for the interpretation of these data, it has to be considered that the knockout mice may have altered gene-expression in order to compensate for the loss of SLC10A4, as shown by an up-regulation of the nicotinic acetylcholine receptor subunits alpha1 and delta in the *Slc10a4* knockout mice. The authors came to the conclusion that the loss of SLC10A4 may result in reduced vesicular filling with the neurotransmitter acetylcholine at the neuromuscular junction [17]. In a further study by Larhammar et al. [15], the *Slc10a4* knockout mice were not different from their wild-type littermates in a series of behavior tests, but showed a hypoactive phenotype. Furthermore, the *Slc10a4* knockout mice were hypersensitive to the psychostimulants amphetamine and tranylcypromine. In the central nervous system, the *Slc10a4* knockout mice had reduced levels of dopamine, serotonin, norepinephrine and choline. Although no evidence for the direct transport of these neurotransmitters by SLC10A4 has been provided, synaptic vesicles from transgenic mice overexpressing the SLC10A4 protein showed an increased uptake of [^3^H]dopamine, which was probably due to higher synaptic vesicle acidification. Based on these data, the authors speculate that SLC10A4 may transport any hitherto unknown organic anion compound, which would allow the vesicular accumulation of higher amounts of protons, which then would increase the vesicular storage of neurotransmitters [15]. This conclusion is in agreement with data from the present study, in which SLC10A4 showed no transport activity for a series of neurotransmitters. However, even suggested organic anion modulators such as ATP or DHEAS were not transported by SLC10A4, meaning that the function of SLC10A4 as solute carrier is still a matter of speculation.

Based on their findings, the Kullander group suggested renaming SLC10A4 as “vesicular aminergic-associated transporter” (VAAT). However, as the naming of the carriers of the SLC10 carrier family until now has only been based on their molecular transport function, i.e. Na^+^/taurocholate co-transporting polypeptide NTCP (*SLC10A1*), apical sodium-dependent bile acid transporter ASBT (*SLC10A2*), and sodium-dependent organic anion transporter SOAT (*SLC10A6*), we strongly suggest not renaming SLC10A4 before its molecular transport function has been elucidated.

The outstanding role of SLC10A4 within the SLC10 family is not only based on its inability to transport bile acids or steroid sulfates. In contrast to NTCP, ASBT and SOAT, which all are sorted to the plasma membrane, SLC10A4 has a typical intracellular vesicle-like expression pattern in neurons, mast cells and neuronal cell lines. This specific expression pattern of SLC10A4 was also observed in the present study in untreated and differentiated CAD and SH-SY5Y cells. To more closely analyze the sorting domains of SLC10A4, a series of NTCP/SLC10A4 chimeras were generated and analyzed in HEK293 and CAD cells. These experiments clearly showed that the cytoplasmic C-terminus of SLC10A4 must contain dominant signals for its vesicular sorting. Previous studies with rat NTCP demonstrated that the tyrosine-based sorting motifs *YXXØ* within its C-terminus (i.e. Y^307^-E-K-I and Y^321^-K-A-A) are involved in membrane delivery [29]. Although the C-terminus of SLC10A4 also contains these potential tyrosine-based sorting motifs (Y^406^-K–K-L und Y^419^-G-T-V), SLC10A4 is obviously not directed to the plasma membrane. In the same way, all chimeric constructs with the C-terminus of SLC10A4 (i.e. NTCP-CtSLC10A4 and NtSLC10A4-NTCP-CtSLC10A4) showed a vesicular expression pattern and failed to transport typical NTCP substrates such as taurocholic acid and DHEAS. In contrast, both chimeras bearing the C-terminus of NTCP (i.e. SLC10A4-CtNTCP and NtNTCP-SLC10A4-CtNCTP) were detected at the plasma membrane. Further mutagenesis experiments now have to localize the vesicular sorting motifs of SLC10A4 at the amino acid level.

## Conclusions

SLC10A4 is expressed in vesicular structures not only in neurons of the central and peripheral nervous system, but also in neuronal cell lines such as SH-SY5Y and CAD. Although different kinds of assays were applied to screen for a transport function, SLC10A4 failed to show transport activity for dopamine, serotonin, norepinephrine, histamine, acetylcholine, choline, acetate, aspartate, glutamate, GABA, PREGS, DHEAS, E-3-S, and ATP, indicating that SLC10A4 does not seem to be a typical neurotransmitter transporter. When the C-terminus of SLC10A4 was replaced by the homologous sequence of NTCP, the SLC10A4-CtNTCP chimera revealed clear plasma membrane expression in CAD and HEK293 cells. Vice versa, the C-terminus of SLC10A4 directed NTCP to intracellular vesicles, indicating that the sorting motifs of both carriers seem to be localized in the C-terminus. Further SLC10A4 transport studies should involve vesicle preparations from SLC10A4-deleted neuronal cell cultures or may use liposomal reconstitution of the SLC10A4 protein. Until then, the functional properties of the SLC10A4 orphan carrier protein still remain unknown.

## Methods

### Materials, chemicals, and radiochemicals

All of the chemicals, unless otherwise stated, were from Sigma-Aldrich (Taufkirchen, Germany). Citalopram was purchased from Biotrend (Cologne, Germany) and collagenase D was from Serva (Heidelberg, Germany). [^3^H]DHEAS (70.5 Ci/mmol), [^3^H]E-3-S (45.6 Ci/mmol), [^3^H]aspartate (11.3 Ci/mmol), [^3^H]GABA (76.2 Ci/mmol), [^3^H]histamine (13.4 Ci/mmol), [^3^H]choline chloride (66.7 Ci/mmol), [^3^H]norepinephrine (56.6 Ci/mmol), [^3^H]serotonin (28.25 Ci/mmol), [^3^H]dopamine (38.7 Ci/mmol), [^3^H]glutamate (49.6 Ci/mmol), [^3^H]ATP (30.9 Ci/mmol), [^35^S]Adenosine 5′-(γ-thio) triphosphate (12.5 mCi/mmol) and [^3^H]acetylcholine iodide (99.7 Ci/mmol) were purchased from PerkinElmer Life Sciences (Boston, MA, USA). [^3^H]PREGS (20 Ci/mmol), [^3^H]taurocholic acid (10.0 Ci/mmol), [^3^H]acetate (150 mCi/mmol), and [^3^H]lithocholic acid (50 Ci/mmol) were obtained from American Radiolabeled Chemicals (St. Louis, MO, USA).

### Culture and differentiation of neuronal cell lines

Human neuroblastoma SH-SY5Y cells (obtained from DSMZ Braunschweig, Germany) were maintained at 37°C in RPMI medium (Gibco, Karlsruhe, Germany) containing 10% fetal calf serum (FCS, Sigma-Aldrich) and 1% penicillin/streptomycin (P/S, containing 100 U/ml penicillin and 100 mg/ml streptomycin). For differentiation, the cultures at an approximate confluence of 40% were placed in serum-free RPMI medium supplemented with either 10 µM *all*-*trans* RA (Sigma-Aldrich) plus 10 ng/ml BMP-2 (BioCat, Heidelberg, Germany) or 10 ng/ml TGF-β1 (Invitrogen, Karlsruhe, Germany) and were grown for 5 days. As a control, cells were grown in serum-free RPMI medium without any supplements. Mouse catecholaminergic CAD cells (obtained from European Collection of Cell Cultures ECACC, Health Protection Agency, UK) were maintained at 37°C in Dulbecco’s modified Eagle’s/Ham’s F-12 (1:1) medium (DMEM/F12) containing 8% FCS, 4 mM l-glutamine and 1% P/S at 37°C. For cell differentiation, 4 × 10^5^ cells were seeded in 6-well or 24-well plates on poly-l-lysine-coated glass coverslips and grown in serum-free medium for 24-72 h. Under these conditions, the cultures stopped proliferating and large neurite-like cell extensions appeared.

### Immunofluorescence analysis of SH-SY5Y, CAD, and HEK293 cells

For immunofluorescence analysis, SH-SY5Y, CAD, and HEK293 cells were seeded at a density of 5 × 10^4^ cells per cm^2^ in 24-well plates on poly-l-lysine-coated glass coverslips and cultured to a confluence of approximately 40%. For immunofluorescence experiments, the cells were washed with phosphate buffered saline (PBS, containing 137 mM NaCl, 2.7 mM KCl, 1.5 mM KH_2_PO_4_, and 7.3 mM Na_2_HPO_4_, at pH 7.4) and fixed with 2% paraformaldehyde (PFA, Roth, Karlsruhe, Germany) in PBS for 15 min at 4°C. Then, cells were again washed with PBS and incubated with 20 mM glycine in PBS for 5 min. For permeabilization, cells were incubated for 5 min with PBT buffer (0.2% Triton X-100 and 20 mM Glycine in PBS). The non-specific binding sites were blocked with 1% bovine serum albumin (BSA) plus 4% goat serum (Sigma-Aldrich) in PBS for 30 min at room temperature. Then, the cells were incubated with the primary antibodies, rabbit anti-Slc10a4 (antibody 1338 C, 1:1,000 dilution, see [13, 14] ), Sigma Prestige rabbit anti-SLC10A4 (1:500 dilution) [Sigma-Aldrich Cat# HPA028835 RRID:AB_10603025], Abnova rabbit anti-Slc10a4 (1:500 dilution) [Abnova Corporation Cat# PAB14855 RRID:AB_10696081], or Abgent rabbit anti-SLC10A4 (1:500 dilution) [Abgent Cat# AP10250b RRID:AB_10821224], or with mouse anti-V5 monoclonal antibody (1:5,000 dilution) [Invitrogen Cat# R96025 RRID:AB_159313] in blocking solution overnight at 4°C. The next day, cells were washed with PBS and incubated with the fluorophore-labeled secondary antibody Cy3-conjugated goat anti-rabbit IgG (1:800 dilution) [Jackson ImmunoResearch Cat# 111-165-003 RRID:AB_2338000] or Alexa fluor 488-labelled goat-anti mouse (1:800 dilution) [Molecular Probes (Invitrogen) Cat# A11001 RRID:AB_141367] in blocking solution for 60 min at room temperature. After several washing steps with PBS, the cells were covered with a DAPI/methanol solution containing 1 µg/ml DAPI (Roche, Mannheim, Germany) and incubated for 5 min at room temperature. The cells were rinsed with methanol, air dried and mounted onto slides with ProLong Gold Antifade (Invitrogen) mounting medium.

### Real-time quantitative PCR analysis

Relative expression analysis for SLC10A4/Slc10a4 was performed with ABI PRISM 7300 technology (Applied Biosystems, Darmstadt, Germany). RNA was isolated from human SH-SY5Y and mouse CAD cells with TriReagent (Sigma-Aldrich) and cDNA was reverse-transcribed using the SuperScript III First Strand Synthesis System (Invitrogen). PCR amplification was achieved with the TaqMan Gene Expression Assays Hs00293728 for human SLC10A4 and Mm00557788 for mouse Slc10a4 (Applied Biosystems). The expression data of human glyceraldehyde 3-phosphate dehydrogenase (GAPDH, Hs99999905) and mouse beta-actin (Mm00607939) were used as an endogenous control. Triplicate determinations were performed in a 96-well optical plate for each target, using 5 µl cDNA, 1.25 µl TaqMan Gene Expression Assay, 12.5 µl TaqMan Universal PCR Master Mix (Applied Biosystems) and 6.25 µl water in each 25 µl reaction. The plates were heated for 10 min at 95°C, and 40 cycles of 15 s at 95°C and 60 s at 60°C were applied. The relative expression (ΔC_T_) of each target was calculated by subtracting the signal threshold cycle (C_T_) of the endogenous control from the C_T_ value of the target.

### Western blot analysis

Protein contents of CAD cell lysates were determined with the BCA Protein Assay Kit (Novagen, Darmstadt, Germany). Samples of 20 µg protein were mixed with Laemmli Sample Buffer (Sigma-Aldrich), separated on a 12% SDS polyacrylamide gel and transferred to a Hybond-ECL nitrocellulose membrane (Amersham Biosciences, Freiburg, Germany). The blotted membranes were blocked with blocking solution containing 5% ECL-blocking agent (Amersham Biosciences) in TBS-T (137 mM NaCl, 10 mM Tris, pH 8.0, 0.05% Tween-20), followed by overnight exposure to antigen-specific primary antibodies at 4°C in the same buffer. After several washing steps in TBS-T, the membranes were probed with the appropriate horseradish-peroxidase-labeled secondary antibodies in TBS-T for 60 min at room temperature. Signals were developed using the Roti-Lumin ECL Detection Kit (Roth) and visualized by exposure to Hyperfilm ECL (Amersham Biosciences).

### Cloning of the reference carriers

Full length transcripts covering the whole open reading frame for human SLC10A4, DAT, CHT1, SERT, VMAT2, VAChT, NTCP, and mouse Oct1 were amplified by PCR with the primers listed in Table 3. All amplicons were sequence-verified by DNA sequencing according to the GenBank Accession Nos. given in Table 3 and were cloned into the pcDNA5/FRT/V5-His TOPO vector (Life Technologies) via T/A cloning.

**Table 3:** Primers used for full-length carrier cloning

Carrier	Forward primer	Reverse primer	GenBank Accession Nos.
Human SLC10A1 (Na^+^/taurocholate co-transporting polypeptide, NTCP)	5′-tct cta gag gat gga ggc cca caa c-3′	5′-ggc tgt gca agg gga gca gtc-3′	[GenBank:NM_003049]
Human SLC10A4	5′-acc gac ggg cag aac gac-3′	5′-gag aga agt ctg agc ggt ttc-3′	[GenBank:NM_152679]
Human SLC6A3 (dopamine transporter, DAT)	5′-ctc cca gtg tgc cca tga gta aga g-3′	5′-cac ctt gag cca gtg gcg gag-3′	[GenBank:NM_001044]
Human SLC5A7 (choline transporter, CHT1)	5′-aaa aat ggc ttt cca tgt gga agg-3′	5′-ctg taa att atc ttc agt ccc ag-3′	[GenBank:NM_021815]
Human SLC6A4 (serotonin transporter, SERT)	5′-agg atg gag acg acg ccc ttg aat tc-3′	5′-cac agc att caa gcg gat gtc ccc a-3′	[GenBank:NM_001045]
Human SLC18A2 (vesicular monoamine transporter, VMAT2)	5′-gcc atg gcc ctg agc gag ctg-3′	5′-gtc act ttc aga ttc ttc atc ttc acc tat c-3′	[GenBank:NM_003054]
Human SLC18A3 (vesicular acetylcholine transporter, VAChT)	5′-cgg aag agc atc ggg gtg-3′	5′-gct gcg ggt gta gta gta g-3′	[GenBank:NM_003055]
Mouse Slc22a1 (organic cation transporter, Oct1)	5′-att tca agc cac cgc agt tc-3′	5′-ggt atg tgg gga ttt gcc t-3′	[GenBank:NM_009202]

### Generation of the SLC10A4/NTCP chimeras

For generation of the SLC10A4/NTCP chimeric constructs, the full-length SLC10A4-cDNA5 and NTCP-pcDNA5 clones with C-terminal V5-tag were used. *Bmt*I and *Hin*dIII restriction sites were introduced in the N-termini (amino acid positions A6 in the NTCP and A78 in the SLC10A4) and C-termini (amino acid positions E296 in the NTCP and E374 in the SLC10A4), respectively, by site-directed mutagenesis, as previously described in detail [8]. Then, by double digestion and re-ligation of the appropriate fragments, the following chimeric constructs were generated and sequence-verified by direct DNA sequencing: SLC10A4-CtNTCP, NtNTCP-SLC10A4-CtNTCP, NtSLC10A4-NTCP, NtSLC10A4-NTCP-CtSLC10A4, and NTCP-CtSLC10A4. For generation of the N-terminally truncated 75ΔSLC10A4 construct, the SLC10A4 open reading frame starting from amino acid 75 was PCR-amplified and cloned, whereby the codon for Gly75 was replaced by an artificial start codon ATG.

### Transfection of HEK293 and CAD cells

HEK293 cells were grown in DMEM supplemented with 10% FCS, 4 mM glutamine and 1% P/S. HEK293 and CAD cells were grown at 37°C in 5% CO_2_. For transient transfection, both cell lines were seeded in 24-well plates at a density of 1.4–2.0 × 10^5^ cells per well and transfection was performed by using the Lipofectamine 2000 reagent according to the manufacturer’s instructions (Invitrogen). For the establishment of stably transfected cells, Flp-In HEK293 cells were used (Invitrogen), as described previously [3]. Briefly, Flp-In HEK293 cells were seeded in 6-well plates coated with poly-d-lysine at a density of 1 × 10^6^ cells per well and grown to 60–80% confluence in antibiotic free medium. Then, the cells were transfected with 1 µg of the respective pcDNA5/FRT/V5-His vector construct plus 7 µg of pOG44 vector. For the selection of positive clones, hygromycin B was used at a concentration of 150 µg/ml.

### Transport studies in HEK293 and CAD cells

For transport studies, 24-well plates were coated with poly-d-lysine for better attachment of the cells. Stably or transiently transfected cells were plated at a density of 1.25 × 10^5^ cells per well and grown under standard medium for 48–72 h. Before starting the transport experiments, cells were washed three times with PBS and pre-incubated with sodium transport buffer (142.9 mM NaCl, 4.7 mM KCl, 1.2 mM MgSO_4_, 1.2 mM KH_2_PO_4_, 1.8 mM CaCl_2_, and 20 mM HEPES, adjusted to pH 7.4). When transport assays were performed in sodium-free transport buffer, sodium chloride was substituted with equimolar concentrations of choline chloride and for the transport assay with the substrate [^3^H]choline chloride it was substituted with equimolar concentrations of lithium chloride. For transport studies with [^3^H]serotonin, the uptake buffer contained 100 µM ascorbic acid and 100 µM pargyline, to prevent the degradation of serotonin. Transport studies were performed by incubating the cells with 250 µl transport buffer containing the radiolabeled and non-radiolabeled compounds for the indicated time at 37°C. CAD cells could not be washed before starting the transport experiments because these cells easily detached after repeated handling. Therefore, these cells were incubated in 250 µl fresh medium plus 50 µl transport buffer containing the radiolabeled and non-radiolabeled compounds. Uptake studies were stopped by removing the transport buffer and washing the cells five times with ice-cold PBS. Afterwards, cells were lysed in 1 N NaOH with 0.1% SDS and the cell-associated radioactivity was measured by liquid scintillation counting. The protein content was determined using aliquots of the lysed cells with BSA as the standard [30].

### Transport studies in permeabilized HEK293 cells

Stably transfected HEK293 cells expressing SLC10A4, VMAT2 or VAChT were seeded in 24-well plates and were grown until confluence. Then, cells were rinsed three times with potassium-rich buffer (PB) containing 110 mM potassium tartrate, 5 mM glucose, 0.2% bovine serum albumin, 200 µM CaCl_2,_ 1 mM ascorbic acid, 10 µM pargyline and 20 mM piperazine-N,N′-bis(2-ethanesulfonic acid) (PIPES), adjusted to pH 6.8. The cells were permeabilized with 15 µM digitonin in PB for 15 min at 37°C, as reported [23]. The buffer was replaced by 250 µl of PB containing the radiolabeled and non-radiolabeled compounds. After incubation for the indicated time at 37°C, the uptake was stopped, as described above.

### Transport studies in *Xenopus laevis* oocytes

The pcDNA5/FRT/V5-His constructs containing the cloned cDNAs were linearized with *Kpn*I. After phenol/chloroform extraction, the mMESSAGEmMACHINE Kit (Ambion, Life Technologies) was used to generate a capped cRNA and the Poly(A) Tailing Kit (Ambion, Life Technologies) was used to add a poly(A) tail to the RNA transcripts. Afterwards, the cRNA was purified with the MEGAclear Kit (Ambion, Life Technologies) according to the manufacturer’s protocol. Oocytes were obtained from female *X. laevis* frogs and incubated in Ca^2+^-free OR-2 solution (82.5 mM NaCl, 5 mM HEPES–NaOH, pH 7.6, 2.5 mM KCl, 1 mM MgCl_2_ and 1 mM Na_2_HPO_4_) supplemented with 0.4 mg/ml collagenase type D (Serva) at 18°C overnight. Then, oocytes were further incubated in modified Barth’s solution (88 mM NaCl, 15 mM HEPES–NaOH, pH 7.6, 2.4 mM NaHCO_3_, 1 mM KCl, 0.3 mM Ca (NO_3_)_2_, 0.41 mM CaCl_2_ and 0.82 mM MgSO_4_) containing 50 µg/ml gentamicin. Defolliculated oocytes were selected and microinjected with 4.6 ng (46 nl) cRNA encoding for the carrier protein or with a corresponding volume of water. After 3 days of culture in modified Barth’s medium, uptake of the indicated radiolabelled substrates was assessed at 25°C in transport buffer containing 100 mM NaCl, 2 mM KCl, 1 mM CaCl_2_, 1 mM MgCl_2_, and 10 mM HEPES-Tris, pH 7.5. After washing with the same buffer, each individual oocyte was dissolved in 500 µl of 10% SDS. The radioactivity was counted after the addition of 4 ml scintillation fluid in a liquid scintillation counter.

### Statistics

Columns are shown as mean ± SD. Prism software (GraphPad Software Inc., San Diego, CA, USA) was used for data presentation and statistical analysis. Statistical significance of two groups was analyzed by Student’s *t* test. Statistical analysis of more than two groups was performed by one-way analysis of variance (ANOVA) followed by Bonferroni post hoc testing.

## Abbreviations

ASBT: Apical sodium-dependent bile acid transporter
ATP: adenosine triphosphate
BMP-2: bone morphogenetic protein 2
BSA: bovine serum albumin
CHT1: choline transporter
DAT: dopamine transporter
DHEAS: dehydroepiandrosterone sulfate
E-3-S: estrone-3-sulfate
FCCP: carbonylcyanid-p-trifluoromethoxyphenylhydrazon
FCS: fetal calf serum
GABA: gamma-aminobutyric acid
HC-3: hemicholinium-3
HEK293: human embryonic kidneys 293 cells
NTCP: Na^+^/taurocholate co-transporting polypeptide
Oct1: organic cation transporter 1
PB: potassium-rich buffer
PBS: phosphate buffered saline
PFA: paraformaldehyde
PREGS: pregnenolone sulfate
RA: retinoic acid
RFP: red fluorescent protein
SERT: serotonin transporter
SLC10: solute carrier family 10
SOAT: sodium-dependent organic anion transporter
TGF-β: tumor growth factor beta
VAChT: vesicular acetylcholine transporter
VMAT2: vesicular monoamine transporter

## References

[1] Geyer J, Wilke T, Petzinger E (2006). The solute carrier family SLC10: more than a family of bile acid transporters regarding function and phylogenetic relationships. Naunyn Schmiedebergs Arch Pharmacol.

[2] Döring B, Lütteke T, Geyer J, Petzinger E (2012). The SLC10 carrier family: transport functions and molecular structure. Curr Top Membr.

[3] Geyer J, Döring B, Meerkamp K, Ugele B, Bakhiya N, Fernandes CF (2007). Cloning and functional characterization of human sodium-dependent organic anion transporter (SLC10A6). J Biol Chem.

[4] Fietz D, Bakhaus K, Wapelhorst B, Grosser G, Günther S, Alber J (2013). Membrane transporters for sulfated steroids in the human testis-cellular localization, expression pattern and functional analysis. PLoS One.

[5] Grosser G, Fietz D, Günther S, Bakhaus K, Schweigmann H, Ugele B (2013). Cloning and functional characterization of the mouse sodium-dependent organic anion transporter Soat (Slc10a6). J Steroid Biochem Mol Biol.

[6] Splinter PL, Lazaridis KN, Dawson PA, LaRusso NF (2006). Cloning and expression of SLC10A4, a putative organic anion transport protein. World J Gastroenterol.

[7] Fernandes CF, Godoy JR, Döring B, Cavalcanti MC, Bergmann M, Petzinger E (2007). The novel putative bile acid transporter SLC10A5 is highly expressed in liver and kidney. Biochem Biophys Res Commun.

[8] Godoy JR, Fernandes C, Döring B, Beuerlein K, Petzinger E, Geyer J (2007). Molecular and phylogenetic characterization of a novel putative membrane transporter (SLC10A7), conserved in vertebrates and bacteria. Eur J Cell Biol.

[9] Visser WE, Wong WS, van Mullem AAA, Friesema ECH, Geyer J, Visser TJ (2010). Study of the transport of thyroid hormone by transporters of the SLC10 family. Mol Cell Endocrinol.

[10] Bijsmans ITGW, Bouwmeester RAM, Geyer J, Faber KN, van de Graaf SFJ (2012). Homo- and hetero-dimeric architecture of the human liver Na^+^-dependent taurocholate co-transporting protein. Biochem J.

[11] Jiang L, Alber J, Wang J, Du W, Yang X, Li X (2012). The Candida albicans plasma membrane protein Rch1p, a member of the vertebrate SLC10 carrier family, is a novel regulator of cytosolic Ca^2+^ homoeostasis. Biochem J.

[12] Alber J, Jiang L, Geyer J (2013). CaRch1p does not functionally interact with the high-affinity Ca^2+^ influx system (HACS) of Candida albicans. Yeast.

[13] Geyer J, Fernandes CF, Döring B, Burger S, Godoy JR, Rafalzik S (2008). Cloning and molecular characterization of the orphan carrier protein Slc10a4: expression in cholinergic neurons of the rat central nervous system. Neuroscience.

[14] Burger S, Döring B, Hardt M, Beuerlein K, Gerstberger R, Geyer J (2011). Co-expression studies of the orphan carrier protein Slc10a4 and the vesicular carriers VAChT and VMAT2 in the rat central and peripheral nervous system. Neuroscience.

[15] Larhammar M, Patra K, Blunder M, Emilsson L, Peuckert C, Arvidsson E (2014). SLC10A4 Is a vesicular amine-associated transporter modulating dopamine homeostasis. Biol Psychiatr.

[16] Zelano J, Mikulovic S, Patra K, Kühnemund M, Larhammar M, Emilsson L (2013). The synaptic protein encoded by the gene Slc10A4 suppresses epileptiform activity and regulates sensitivity to cholinergic chemoconvulsants. Exp Neurol.

[17] Patra K, Lyons DJ, Bauer P, Hilscher MM, Sharma S, Leão RN (2014). A role for solute carrier family 10 member 4, or vesicular aminergic-associated transporter, in structural remodelling and transmitter release at the mouse neuromuscular junction. Eur J Neurosci.

[18] Abe T, Kanemitu Y, Nakasone M, Kawahata I, Yamakuni T, Nakajima A (2013). SLC10A4 is a protease-activated transporter that transports bile acids. J Biochem.

[19] Gómez-Santos C, Ambrosio S, Ventura F, Ferrer I, Reiriz J (2002). TGF-β1 increases tyrosine hydroxylase expression by a mechanism blocked by BMP-2 in human neuroblastoma SH-SY5Y cells. Brain Res.

[20] Kohr D, Tschernatsch M, Schmitz K, Singh P, Kaps M, Schäfer KH (2009). Autoantibodies in complex regional pain syndrome bind to a differentiation-dependent neuronal surface autoantigen. Pain.

[21] Qi Y, Wang JK, McMillian M, Chikaraishi DM (1997). Characterization of a CNS cell line, CAD, in which morphological differentiation is initiated by serum deprivation. J Neurosci.

[22] Geyer J, Godoy JR, Petzinger E (2004). Identification of a sodium-dependent organic anion transporter from rat adrenal gland. Biochem Biophys Res Commun..

[23] Erickson JD, Eiden LE, Hoffman BJ (1992). Expression cloning of a reserpine-sensitive vesicular monoamine transporter. Proc Natl Acad Sci USA.

[24] Rupprecht R (1997). The neuropsychopharmacological potential of neuroactive steroids. J Psychiatr Res.

[25] Reddy DS (2003). Pharmacology of endogenous neuroactive steroids. Crit Rev Neurobiol.

[26] Zheng P (2009). Neuroactive steroid regulation of neurotransmitter release in the CNS: action, mechanism and possible significance. Prog Neurobiol.

[27] Jiang Y, Wu J, Hua Y, Keep RF, Xiang J, Hoff JT (2002). Thrombin-receptor activation and thrombin-induced brain tolerance. J Cereb Blood Flow Metab.

[28] Goldberg AA, Beach A, Davies GF, Harkness TAA, Leblanc A, Titorenko VI (2011). Lithocholic bile acid selectively kills neuroblastoma cells, while sparing normal neuronal cells. Oncotarget.

[29] Sun AQ, Arrese MA, Zeng L, Swaby I, Zhou MM, Suchy FJ (2001). The rat liver Na^+^/bile acid cotransporter. Importance of the cytoplasmic tail to function and plasma membrane targeting. J Biol Chem.

[30] Lowry OH, Rosebrough NJ, Farr AL, Randall RJ (1951). Protein measurement with the Folin phenol reagent. J Biol Chem.

